# Pendulum-Type Hetero-Core Fiber Optic Accelerometer for Low-Frequency Vibration Monitoring

**DOI:** 10.3390/s18082528

**Published:** 2018-08-02

**Authors:** Hiroshi Yamazaki, Ichiro Kurose, Michiko Nishiyama, Kazuhiro Watanabe

**Affiliations:** 1 Department of Information Systems Science, Faculty of Science and Engineering, Soka University, 1-236 Tangi-machi, Hachioji, Tokyo 192-8577, Japan; e17m5213@soka-u.jp; 2 Department of Science and Engineering for Sustainable Innovation, Faculty of Science and Engineering, Soka University, 1-236 Tangi-machi, Hachioji, Tokyo 192-8577, Japan; mnishiya@soka.ac.jp (M.N.); kazuhiro@soka.ac.jp (K.W.)

**Keywords:** structural health monitoring, fiber optic sensor, accelerometer, hetero-core, low-frequency vibration measurement

## Abstract

In this paper, a novel pendulum-type accelerometer based on hetero-core fiber optics has been proposed for structural health monitoring targeting large-scale civil infrastructures. Vibration measurement is a non-destructive method for diagnosing the failure of structures by assessing natural frequencies and other vibration patterns. The hetero-core fiber optic sensor utilized in the proposed accelerometer can serve as a displacement sensor with robustness to temperature changes, in addition to immunity to electromagnetic interference and chemical corrosions. Thus, the hetero-core sensor inside the accelerometer measures applied acceleration by detecting the rotation of an internal pendulum. A series of experiments showed that the hetero-core fiber sensor linearly responded to the rotation angle of the pendulum ranging within (−6°, 4°), and furthermore the proposed accelerometer could reproduce the waveform of input vibration in a frequency band of several Hz order.

## 1. Introduction

The existence of structural damage and any other faults in civil infrastructures have to be accurately detected early in order to prevent serious accidents caused by infrastructures collapsed for long-term usages, although the number of inspectors is insufficient comparing to the large amount of infrastructures including buildings, bridges, tunnels, and so on. The process of implementing fault diagnosis strategy is referred to as structural health monitoring (SHM), in which the failure in a structure is automatically observed by monitoring, data processing, and health evaluation systems [1].

As one of SHM techniques, fault diagnosis based on vibration monitoring was widely known as a non-destructive sensing and analysis scheme for global fault diagnosis and has been studied so far in the literature [2,3,4,5]. Natural frequency observed in ambient vibration on infrastructures contains the information of structural features so that the transition of the natural frequency implies the deterioration of the structures themselves. Typically, a decrease in stiffness of the structure is observed in a natural frequency getting a lower than expected value. In contrast, a frequency higher than expected implies that the structure was stiffer than expected. Although natural frequency of structures can also fluctuate depending on unmonitored ambient environmental factors such as temperature and humidity, damage detection methodologies excluding environmental effects have been discussed by use of statistical process controls [6]. For instance, Soman et al. employed several stiffness indices based on modal frequency, displacement, and strain for quantifying the degree of structural health by use of a multi-metric measurement system [7]. Moreover, a practical non-destructive bridge condition assessment was examined on an actual bridge using accelerometers in combination with cable tension sensors, anemometers, and thermistors [8]. The relation between modal parameters obtained from mechanical vibration and the extent of damage was also observed in actual bridges in a low frequency band under 20 Hz with statistical evaluation methods [9].

There have been a number of electric-based sensing devices developed using piezoelectric and capacitive sensors [10,11,12,13,14]. However, when considering practical usages in actual infrastructures, it is necessary for the sensor devices to be highly robust to electromagnetic interference, chemical corrosion, and severe temperature changes in order to monitor civil infrastructures for the long-term in harsh environments, which is always exposed to changes in humidity, or sometimes water leakage and lightning damages [15]. In comparison, optical accelerometers have attracted attention as an alternative sensing technology to electric sensors for SHM because of their remarkable merits. Optical accelerometers are non-electric and passive sensor devices, and immune to electromagnetic interference and chemical corrosion. Liu et al. developed an optical intensity-modulated accelerometer for low-frequency vibration monitoring [16,17], in which the displacement of a pendulum was detected by use of a simple optical path design based on the Talbot effect with high dynamic range, whereas the accelerometer is not suitable for long-term remote sensing due to the necessity of power supply to the sensor portion.

On the other hand, fiber optic accelerometers employ optical fiber lines with low transmission loss so as to be suitable for outdoor long-term remote sensing [18]. There have been a variety of fiber optic sensors developed for monitoring the condition of structures by use of Brillouin scattering [19,20], fiber Bragg grating (FBG) [21,22,23,24,25,26,27,28], and optical interferometer [29,30]. Minardo et al. introduced distributed fiber optic temperature and strain sensors using Brillouin optical time-domain analysis (BOTDA) into railway infrastructures and succeeded to dynamically monitor strain distribution due to train passage [19]. Moreover, a considerable number of fiber Bragg grating (FBG) sensors have also been proposed for detecting strain in structures by embedding sensors themselves [21,22,23], and they can perform as accelerometers by means of sensing the deformation of an oscillated cantilever beam [24,25,26]. A fiber optic accelerometer based on Fabry-Perot interference was also investigated [29]. This accelerometer detects vibration in a range up to some hundreds Hz in such a way to measure the flexure of an optical fiber cantilever beam by Fabry-Perot interference.

In this paper, we have proposed a novel accelerometer based on a hetero-core fiber optic sensor for monitoring low-frequency vibration on large-scale infrastructures. Hetero-core fiber optic sensors consist of two single-mode fibers fusion-spliced with different core diameters to obtain high sensitivity to macro-bending on the processed fiber line [31]. Compared to conventional fiber optic sensors, the hetero-core fiber sensors have some attractive features such as a cost-effective intensity-modulated measurement scheme by only use of a set of a light emitting diode (LED) and a photo diode (PD) with an output wavelength of 1.31 μm. Moreover, it has temperature independency at the sensor portion owing to the thermal expansion of silica glass fiber negligibly affecting the curvature of the hetero-core portion with a length of few millimeters.

The scope of this study is to realize a pendulum-type accelerometer targeting low-frequency natural vibration on large-scale infrastructures, in which an embedded hetero-core sensor detects the rotation angle of a pendulum. The movement of the pendulum was subjected to a combination of a weight and springs equipped with the pendulum so that they dominantly determined the resonance characteristics of the proposed accelerometer. On the other hand, it was so far demonstrated that the hetero-core fiber optic sensor served as a displacement sensor with high linearity by a conversion mechanism to transform displacement to macro-bending [32], thereby the rotation angle of the pendulum can be detected in such a way that the hetero-core optical fiber measured the displacement on a given point on the pendulum. A series of experiments were conducted to confirm that the hetero-core optical fiber inside the accelerometer measures the rotation of internal pendulum with a good linearity. In addition, it was revealed that the accelerometer performed well in low-frequency vibration measurement by tuning the sensitivity and frequency response characteristics depending on a mass and a spring coefficient of the pendulum.

## 2. Sensor Principle

**Figure 1:**
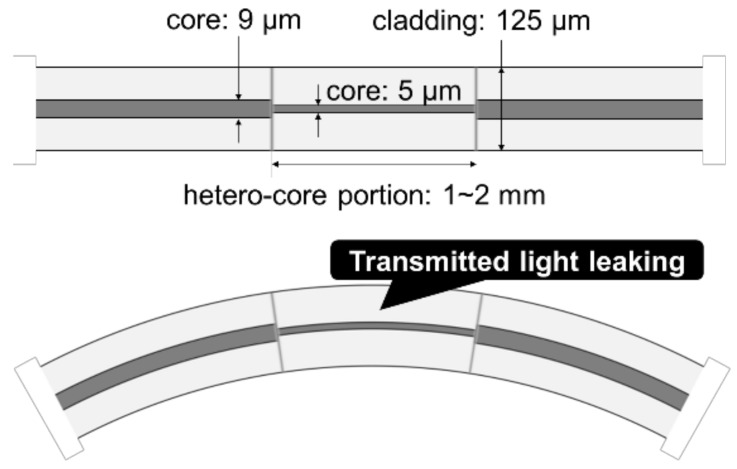
Schematics of a hetero-core fiber optic sensor.

As shown in Figure 1, a hetero-core fiber optic sensor proposed in this paper is composed of a short single mode (SM) fiber segment called a hetero-core portion, inserted by fusion splicing into an SM fiber transmission line. The core diameters of the hetero-core portion and the transmission line are 5 μm and 9 μm, respectively, and the length of the hetero-core portion is about 1–2 mm. It was previously confirmed that light transmitted through the core partially leaks into a cladding layer at a boundary between the transmission fiber and the hetero-core portion, and the degree of light leakage increases with a bending radius of the hetero-core portion [31].

A displacement sensor based on hetero-core fiber optics has been developed in a previous research [32] by employing the conversion mechanism from displacement to bending. This displacement sensor was composed of the hetero-core optical fiber clamped across the hetero-core portion by a pair of fiber clampers, one of which was set to move and the other was fixed. When the displacement of the clampers, d, increases, the optical loss of the sensor was linearly increased as the increment of bending radius on the hetero-core portion. The accuracy of the sensor to the displacement was less than 0.1% FS so that the hetero-core fiber optic sensor can be employed as a highly-accurate displacement sensor.

**Figure 2:**
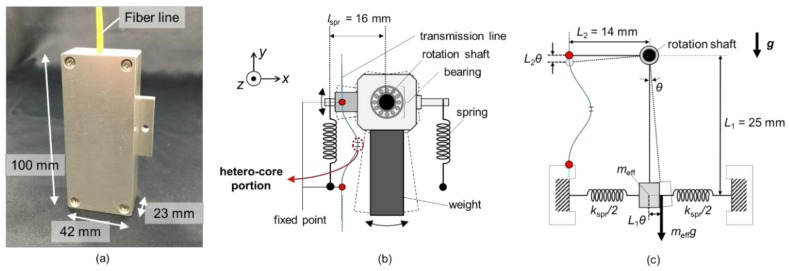
(**a**) Photo of outside appearance; (**b**) internal structure; and (**c**) schematics of a pendulum-type accelerometer with a hetero-core fiber optic sensor.

Figure 2 illustrates schematics of the proposed pendulum-type accelerometer based on hetero-core fiber optics. This accelerometer houses a pendulum and a hetero-core optical fiber with dimensions of 100 mm × 42 mm × 23 mm, as shown in Figure 2a. As can be seen in Figure 2b, a pendulum built in the accelerometer equips a weight and two springs and rotates on a shaft fixed on a chassis of the sensor. A hetero-core optical fiber is clamped at two points, one of which is on the pendulum and the other is fixed on the chassis, thereby the sensor detects the rotation angle of the pendulum as a displacement of the clamped point on the pendulum. In order not to attenuate the movement of pendulum by plastic coating resin on a bending optical fiber, the hetero-core optical fiber was bare between two fixed points. When a whole system of the accelerometer was accelerated in the x-axis direction, the internal pendulum rotates due to an inertial force and displaces the position of the clamped point of the hetero-core fiber. Figure 2c shows the simplified vibration model of the proposed accelerometer, in which it is supposed that the weight of pendulum exists at a centroid of weight (far from the rotation shaft by *L*_1_ = 25 mm) and is physically subjected to two springs. The effective mass of the weight, *m*_eff_, is determined from the mass of the weight, m, those of other components, *m_i_*, and the distance of components from the rotation shaft, *l_i_*, by moment equation as follows:

(1) $$m_{\mathrm{eff}}=m+\frac{1}{L_{1}}\sum_{i}m_{i}l_{i},$$

In addition, the effective elastic coefficient, *k*_eff_, is also expressed from *k*, the elastic coefficient of two employed springs, as follows:

(2) $$k_{\mathrm{eff}}=2k_{\mathrm{spr}}+k_{\mathrm{fib}}\frac{L_{2}}{L_{1}},$$

in which *k*_spr_ is the effective coefficient of springs, which is calculated by use of the spring coefficient, *k*, and the distance of the springs from the rotation shaft, *l*_spr_, expressed as $k_{\mathrm{spr}}=k\frac{l_{\mathrm{spr}}}{L_{1}}$. It is assumed that the hetero-core optical fiber also contributes to the system of pendulum as an elastic component with a spring coefficient *k*_fib_. Therefore, when the pendulum is rotated by input acceleration *α*(*t*), the equation of motion in the pendulum is described as follows:

(3) $$m_{\mathrm{eff}}L_{1}\ddot{\theta }+\lambda \left(\dot{\theta }\right)+k_{\mathrm{eff}}L_{1}\theta +m_{\mathrm{eff}}g\theta =m_{\mathrm{eff}}\alpha \left(t\right),$$

where *θ* denotes a rotation angle of the pendulum and *λ* represents a damping factor of the system including friction around the rotation shaft. The pendulum is also subject to a component of gravity parallel to the rotation motion, as described as a fourth term in the left side of Equation (3). From this equation, the resonant frequency of this vibration system, *f*_0_, can be derived as follows:

(4) $$f_{0}=\frac{1}{2\pi }\sqrt{\frac{k_{\mathrm{eff}}}{m_{\mathrm{eff}}}+\frac{g}{L_{1}}}.$$

Furthermore, when the frequency of input vibration, *α*(*t*), is sufficiently lower than the resonant frequency, the following relational expression holds for *α* and *θ* because of the first and second terms in the left side of Equation (3) can be neglected.

(5) $$\frac{d\theta }{d\alpha }=\frac{m_{\mathrm{eff}}}{L_{1}k_{\mathrm{eff}}+m_{\mathrm{eff}}g}=\frac{1}{L_{1}{\left(2\pi f_{0}\right)}^{2}}.$$

On the other hand, the hetero-core fiber optic sensor detects the rotation as the displacement *L*_2_*θ*, when *θ* is sufficiently small. The relation between the rotation angle, *θ*, and an optical loss of the hetero-core sensor is described as follows, on the condition that the sensor linearly responds to the displacement:

(6) $$Loss=\gamma^{\prime }L_{2}\theta =\gamma \theta ,$$

in which the coefficient, *γ*, means the ratio of optical loss response to the rotation angle. Therefore, considering Equations (5) and (6), the sensitivity of the accelerometer can be obtained by the following formula:

(7) $$\frac{dLoss}{d\alpha }=\frac{\gamma }{L_{1}{\left(2\pi f_{0}\right)}^{2}}.$$

It can be seen from Equation (7) that the sensitivity has trade-off relation to the resonant frequency which determines the width of a measurable frequency band, a combination of a weight and springs should be modulated in balance for measuring low-frequency natural vibration in infrastructures with high sensitivity.

Let us consider when acceleration is applied to the accelerometer in multi-axis directions. Because the pendulum in the accelerometer was mechanically allowed to move only in the direction of rotation of the bearing, the accelerometer is insensitive to the acceleration applied along the z-axis, as shown in Figure 2b. On the other hand, the centroid of the pendulum is slightly biased to the left because of the fixed part of an optical fiber in the left part of the pendulum, thereby the pendulum slightly rotates due to the acceleration applied along the z-axis. This cross-axis sensitivity is considerably smaller than the sensitivity to the acceleration applied along the x-axis, and furthermore can be lowered by readjusting the centroid of the pendulum to the center in the x-axis direction.

## 3. Static Rotation Response

**Figure 3:**
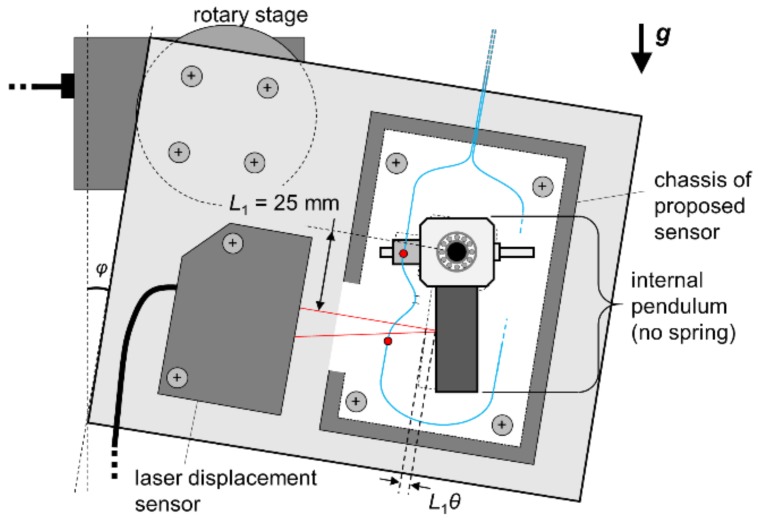
Experimental setup for hetero-core fiber sensitivity to the rotation of a pendulum in the inclined accelerometer.

For evaluating the sensitivity and linearity of a hetero-core fiber optic sensor to the rotation of pendulum, the relation between a rotation angle of the pendulum and optical loss response of the fiber sensor was monitored. As shown in Figure 3, the proposed accelerometer and a laser displacement sensor measuring the centroid position of the pendulum were fixed on a firm plate rotated by a rotary stage. The rotary stage got the plate slowly inclined by *φ* in the range of ±8° with a stepwise of 0.5°, and the optical loss and the displacement of weight were simultaneously recorded. In this experiment, two springs inside the accelerometer were removed in advance in order to reduce the difference between the inclination angle, *φ*, and the internal rotation angle of pendulum, *θ*. Furthermore, the displacement of weight measured by the laser displacement sensor was regarded as *L*_1_*θ* because the rotation angle, *θ*, would be sufficiently small.

**Figure 4:**
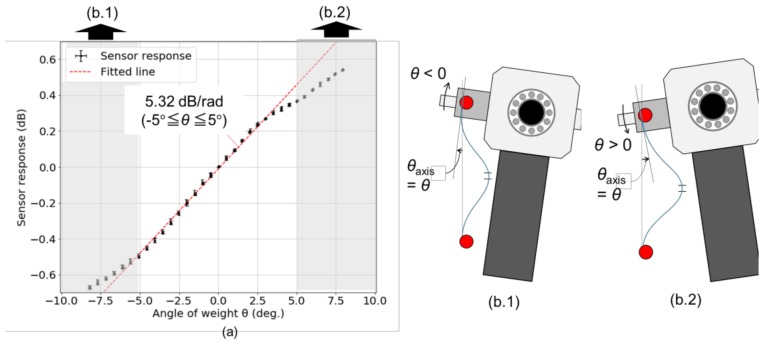
(**a**) Optical loss variation of a hetero-core optical fiber inside a proposed accelerometer as functions of angle of weight, *θ*; and (**b.1**,**b.2**) schematics of excess changes in the axial angle of the optical fiber line, *θ*_axis_, due to the rotation movement of fixed point on the pendulum.

Figure 4a shows the variation of optical loss as a function of the rotation angle of weight, *θ*, measured by the laser displacement sensor. It can be seen that the optical loss linearly changed with a sensitivity of 5.37 dB/rad in the range −6° < *θ* < 4°. In spite of the linear response of a hetero-core fiber optic sensor to the displacement as mentioned in Section 2, nonlinear responses in ranges *θ* < −6° and *θ* > 4° would result from the change of an axial angle of the optical fiber at a fixed point on the pendulum. *θ*_axis_, the axial angle of the optical fiber at a fixed point on the pendulum, was set to *θ*_0_ > 0° in an initial pendulum position (*θ* = 0°) in order to guide the bending direction of hetero-core optical fiber. When the pendulum rotated by *θ* < 0°, as depicted in Figure 4(b.1), *θ*_axis_ was changed to *θ* + *θ*_0_, which made the curvature on the hetero-core portion higher than the case where the only displacement *L*_2_*θ* was applied with *θ*_axis_ = *θ*_0_. Similarly, in the case of *θ* > 0°, as shown in Figure 4(b.2), the change of *θ*_axis_ lowered the increment of curvature on the hetero-core portion due to the applied displacement. Because the absolute amount of *θ*_axis_ was smaller in minus direction than in plus direction due to the initial axial angle *θ*_0_ > 0, the range of linear response biased to the minus direction. Although this phenomenon would always appear in this sensing mechanism, the effect on sensor response appeared to be negligible in the small rotation angle so that the sensitivity of the hetero-core optical fiber to rotation angle, *γ*, is 5.37 dB/rad with a linearity in the range of −6° < *θ* < 4°.

## 4. Frequency Response Characteristics

**Figure 5:**
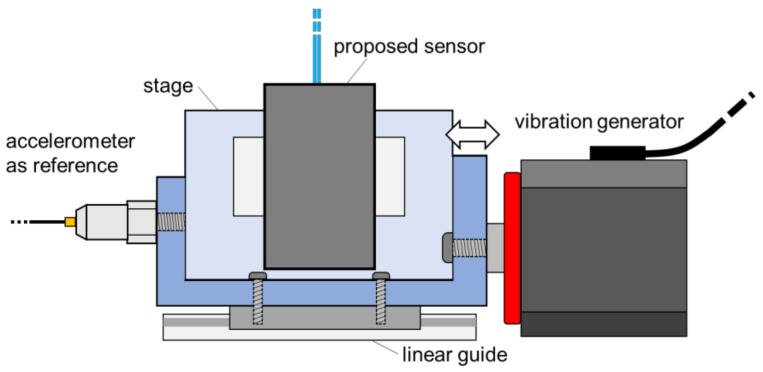
Experimental setup for frequency response characteristics of the proposed accelerometer.

To understand the optimum specification of the proposed accelerometer for monitoring low-frequency vibration, the frequency responses were monitored with arranging combinations of the mass of weight, *m*, and the spring coefficient, *k*, as listed in Table 1. In this experiment, as illustrated in Figure 5, the proposed accelerometer was attached together with a commercial accelerometer (B51C BC46, Asahi Seisakusyo, Tokyo, Japan) used as a reference on a stage which was horizontally vibrated by a vibration generator (WaveMaker01, Asahi Seisakusyo). The waveform applied by the vibration generator was set to be sinusoidal with a frequency ranging from 1 Hz to 50 Hz, and in each frequency both the reference acceleration data and the optical loss responses were simultaneously measured for 10 s by a sampling rate of 4 kHz, respectively.

**Figure 6:**
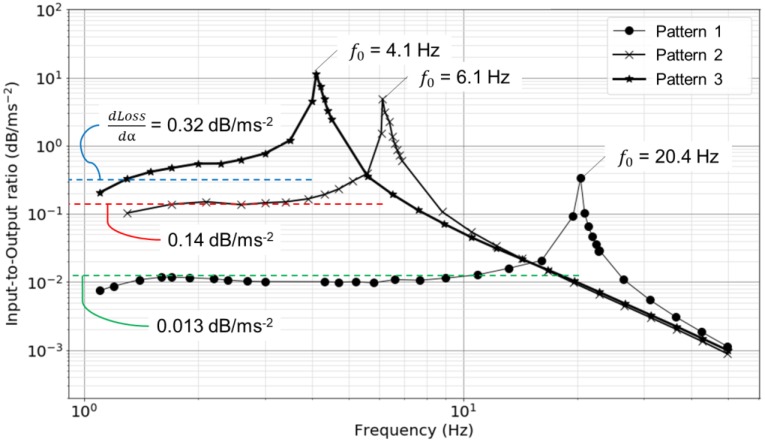
Frequency response characteristics of the proposed accelerometer which was modulated in three patterns shown in Table 1.

Figure 6 shows the frequency response of the proposed accelerometer, which was obtained as an input-to-output ratio by comparing the amplitudes of the fast Fourier transform (FFT) spectra for each frequency. When calculating *dLoss*/*dα* by obtained *f*_0_ according to Equation (7), the values plotted as dashed lines in Figure 6 were found to overlap with the measured in frequency bands lower than *f*_0_. In the case of Pattern 1, the input-to-output ratio seems to gradually increase as the increment of frequency. In the other cases of Patterns 2 and 3, however, the input-to-output ratios were stable within an error of 10% in low-frequency bands of 1.7–3.9 Hz and 1.4–10.9 Hz, respectively. Therefore, the sensitivity of accelerometer in Patterns 2 and 3 can be given as a constant with the accuracy of 10% in the above frequency ranges.

Additionally, the resonant frequency, *f*_0_, and sensitivity, *dLoss*/*dα*, obtained in the experiments were compared to simulated values according to the sensor principle. As mentioned in the Section 2, $\frac{1}{L_{1}}\sum_{i}m_{i}l_{i}$ and *k*_fib_ in Equations (1) and (2) were unknown parameters for determining the simulated resonant frequency, *f*_0′_, so that the two parameters were estimated due to the experimental results of *f*_0_ by fitting all data of *f*_0′_ in three patterns to the experimental data of *f*_0_ for minimizing the errors of *f*_0′_ to *f*_0_ in three patterns. Consequently, it was found that the errors could be within 7% at the most when $\frac{1}{L_{1}}\sum_{i}m_{i}l_{i}$ = 0.018 kg and *k*_fib_ = 2.2 N/m, which are reasonable with respect to the actual dimensions of components, except for a weight and a considerably smaller elasticity of a bending fiber line than springs. Table 1 shows the simulated and experimental values, in which the parameters $\frac{1}{L_{1}}\sum_{i}m_{i}l_{i}$ and *k*_fib_ were set as the above. It was observed that the resonant frequency, *f*_0_, shifted with a tendency like *f*_0′_ described in Table 1, and the sensitivity decreased inversely to the resonant frequency.

**Figure 7:**
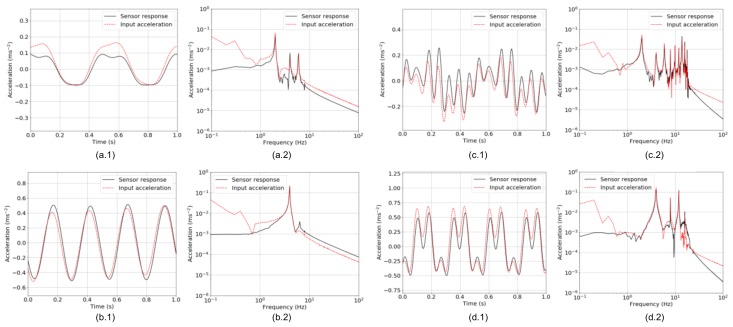
Profiles of responses of the proposed accelerometer and input vibration in (x.1) time domain and (x.2) frequency domain: (**a**) 2-Hz vibration applied to the proposed accelerometer in Pattern 2; (**b**) 4-Hz vibration to the accelerometer in Pattern 2; (**c**) 2-Hz vibration to the accelerometer in Pattern 3; and (**d**) 4-Hz vibration to the accelerometer in Pattern 3.

Figure 7 shows profiles of sensor responses in the cases of Patterns 2 and 3 when 2- and 4-Hz vibrations were applied. In order not to take into consideration vibrations excited due to resonance, high-frequency components were numerically removed by the finite impulse response (FIR) low-pass filtering with a cut-off frequency of 0.8 × *f*_0_ Hz. In the case of Pattern 2, it can be seen in Figure 7(a.1,b.1) that the proposed accelerometer responded to vibration with no phase delay comparing to input acceleration data measured by the reference sensor. Moreover, the frequency spectra depicted in Figure 7(a.2,b.2) indicated that the proposed accelerometer well reproduced the frequency spectrum of input acceleration in the range from 1 Hz to the cut-off frequency. There were multiple peaks observed when 2-Hz vibration was applied, as shown in Figure 7(a.2), which were derived from unwanted harmonic components of input vibration generated by the vibration generator. On the other hand, spectral components under 1 Hz tended to be lower in the sensor response than in input vibration. This would cause the proposed accelerometer to have a reduced sensitivity at around 1 Hz, which can be supposed in Figure 6. These features in terms of a phase shift and a spectral form in a frequency band ranging from 1 Hz to 0.8 × *f*_0_ Hz were similarly observed in the case of Pattern 3, as shown in Figure 7c,d. As a result, it was confirmed that the proposed accelerometer performed well in detecting distinctive spectral peaks on the applied acceleration in a frequency band more than 1 Hz and less than 0.8 × *f*_0_ Hz, with keeping the theoretical trade-off relation between the sensitivity and resonant frequency. In addition, for the accelerometer responding linearly in the rotation angle range of (−6°, 4°), the measurement ranges can be calculated as (−3.9 m·s^−2^, 2.6 m·s^−2^) and (−43.0 m·s^−2^, 28.7 m·s^−2^) in the cases of Patterns 2 and 3, respectively. Because the sensitivity and the width of detectable frequency band had a trade-off relation, the accelerometer should be optimized for detecting representative components of natural-frequency vibration of a target infrastructure in terms of the amplitude and frequency band, with moderate adjustments on the internal weight and springs such as in Patterns 2 and 3.

## 5. Conclusions

This paper described a novel pendulum-type accelerometer based on hetero-core fiber optics for monitoring low-frequency vibrations observed in large-scale infrastructures such as bridges, building, and tunnels. This accelerometer contained a pendulum with a weight and springs, the rotation angle of which was measured by an embedded hetero-core fiber optic sensor. Considering an equation of motion in the system of pendulum, the rotation angle was proportional to applied acceleration in a range under a resonant frequency.

Through a static rotation test, it was found that the hetero-core fiber sensor responded to the rotation angle with a linear sensitivity of 5.37 dB/m·s^2^ in a range within (−6°, 4°), although it nonlinearly responded out of this range because the rotation of a fixed point of a fiber line on the pendulum broke the linearity of the sensor response. Moreover, the ratio of rotation angle to applied acceleration varied inversely with respect to the value of resonant frequency, *f*_0_, and the sensitivity of the accelerometer was able to be calculated from the experimentally observed *f*_0_. Additionally, it was also confirmed that the accelerometer showed stable sensitivities within an error of 10% in low-frequency bands of 1.7–3.9 Hz and 1.4–10.9 Hz when the accelerometer was modulated to have resonant frequency, *f*_0_, of 6.1 Hz and 20.4 Hz, respectively, and the accelerometer reproduced a spectrum waveform of input acceleration in a frequency band from 1 Hz to 0.8 × *f*_0_ Hz. In addition, there was a trade-off relation between the sensitivity and the width of detectable frequency band observed in frequency response characteristics so that the accelerometer should be modulated for the amplitude and frequency of target vibration in infrastructures. Consequently, the findings from these experiments have suggested that the proposed accelerometer was suitable for monitoring vibration in a frequency band of several Hz order, and therefore performed well for monitoring the positions of natural frequencies observed in a low frequency band in large-scale infrastructures.

## Figures and Tables

**Table 1:** The combinations of *m* and *k* of the proposed accelerometer, and simulated and experimental values of resonant frequency and sensitivity.

Accelerometer	*m* (kg)	*k* (N/m)	Simulated Value		Experimental Value	
			${f^{\prime }}_{0}\;(\mathrm{Hz})$	${\frac{dLoss}{d\alpha }}^{\prime }\;(\mathrm{dB}/m\cdot s^{-2})$	$f_{0}\;(\mathrm{Hz})$	$\frac{dLoss}{d\alpha }\;(\mathrm{dB}/m\cdot s^{-2})$
Pattern 1	0.042	16	4.4	0.28	4.1	0.32
Pattern 2	0.0063	16	5.7	0.17	6.1	0.14
Pattern 3	0.0063	350	21.8	0.011	20.4	0.013
